# Duodenal metal stent deployment using a novel, cholangioscope-guided, guidewire insertion technique

**DOI:** 10.1055/a-2183-6315

**Published:** 2023-11-20

**Authors:** Takeshi Ogura, Junichi Nakamura, Jun Sakamoto, Yuki Uba, Hiroki Nishikawa

**Affiliations:** 1 13010 Endoscopy Center, Osaka Medical and Pharmaceutical University, Takatsuki, Japan; 2 13010 2nd Department of Internal Medicine, Osaka Medical and Pharmaceutical University, Takatsuki, Japan

Malignant gastric outlet obstruction (GOO) occurs with advanced or metastatic malignancies located in the duodenum and is present in up to 19% of patients with unresectable malignant tumors 1 . Duodenal obstruction can be traditionally treated by gastrojejunostomy, but endoscopic duodenal stenting has been suggested as a less invasive treatment 2 . More recently, endoscopic ultrasound-guided gastroenterostomy using lumen-apposing metal stents has been reported 3 4 . However, as this technique may need to be performed by experts or in high-volume centers, endoscopic duodenal stenting is still an important procedure. During duodenal stenting, guidewire passage through the stricture to the anal side is needed, but this technique is sometimes challenging. Recently, a novel cholangioscope (eyeMAX; Micro-Tech Co., Ltd., Nanjing, China), which offers improved visibility, has become available. This report describes guidewire deployment using this novel cholangioscope for guidance in duodenal stent deployment in a case of duodenal obstruction.

A 58-year-old man was admitted to our hospital with GOO caused by cancer of the head of the pancreas. This patient had previously undergone biliary drainage using a covered self-expandable metal stent (SEMS). Duodenal stenting was attempted. First, the duodenoscope was advanced into the ampulla of Vater. However, because of the SEMS, the duodenal obstruction site could not be observed endoscopically. Therefore, an endoscopic retrograde cholangiopancreatography catheter was inserted ( Fig. 1 ), and guidewire insertion through the obstruction site was attempted; however, guidewire insertion failed ( Fig. 2 ). A novel cholangioscope was then inserted, and the obstruction site could be observed clearly ( Fig. 3 ). Guidewire insertion was performed successfully under direct visualization ( Fig. 4 ). After duodenography, duodenal metal stent deployment was performed successfully without any adverse events ( Fig. 5 , Video 1 ).

**Fig. 1:**
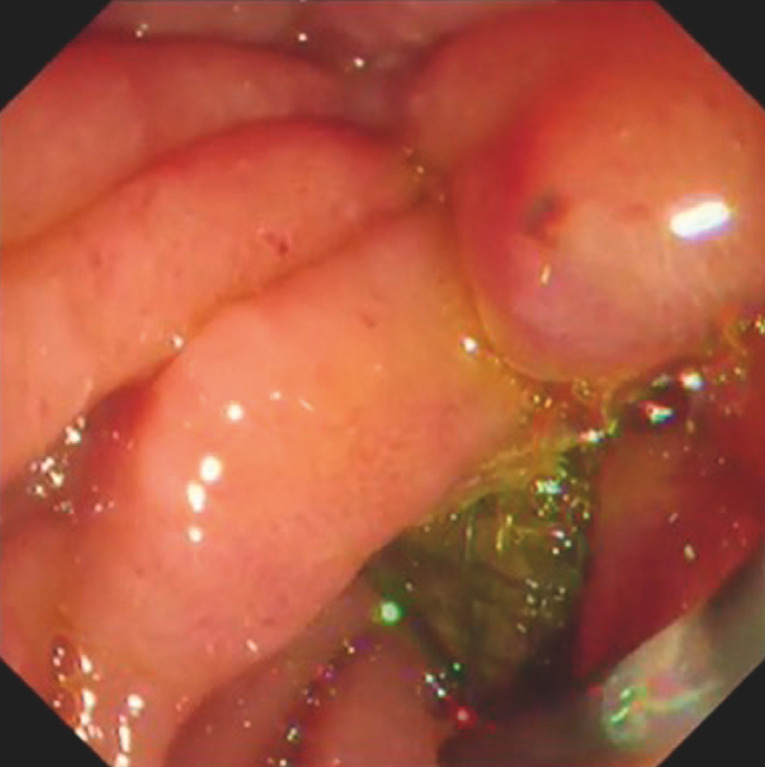
An endoscopic retrograde cholangiopancreatography catheter was inserted because the duodenal obstruction site could not be seen owing to the presence of the biliary stent.

**Fig. 2:**
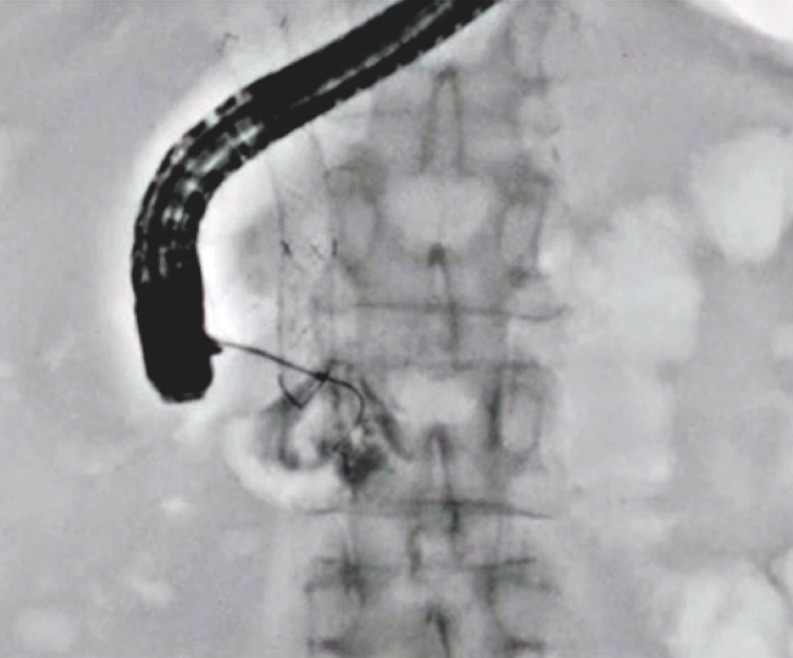
Guidewire insertion failed under fluoroscopic guidance.

**Fig. 3:**
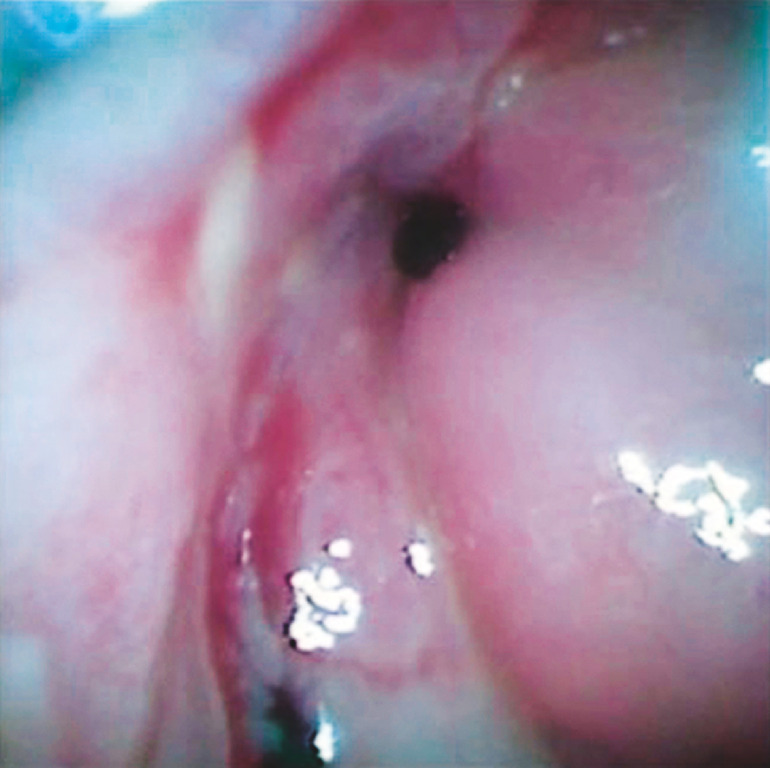
The duodenal obstruction site could be seen clearly with the novel cholangioscope.

**Fig. 4:**
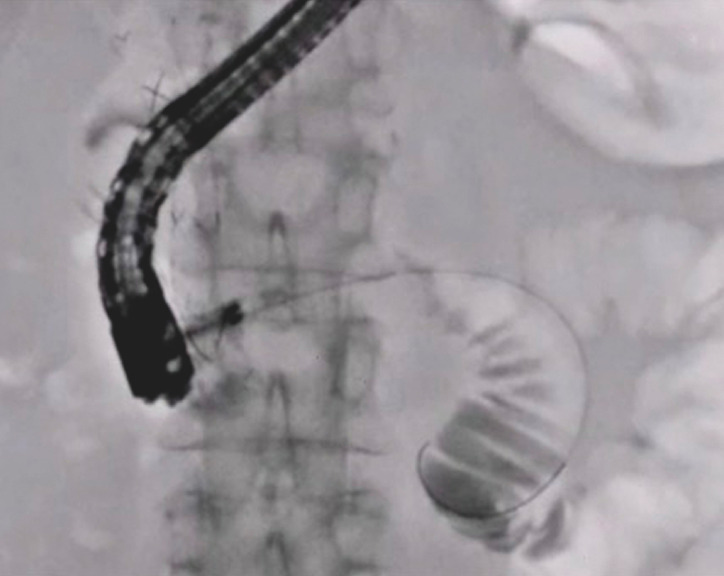
Guidewire insertion was performed successfully.

**Fig. 5:**
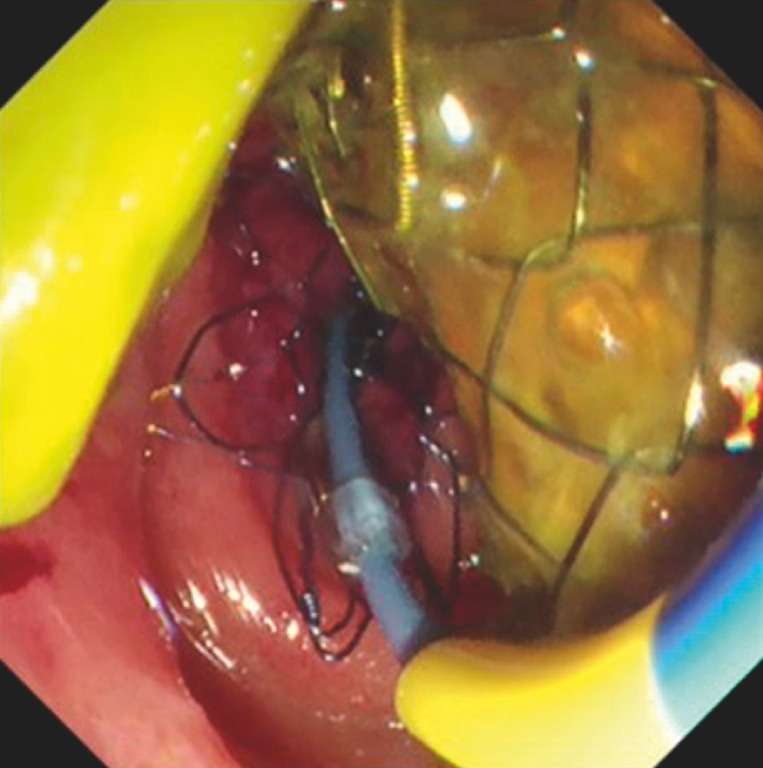
Duodenal stenting was performed successfully.

Guidewire insertion through the duodenal obstruction site was attempted but failed. A novel cholangioscope was inserted, and the obstruction site was observed. Guidewire insertion and duodenal stenting were performed successfully.Video 1

In conclusion, a novel cholangioscope may be useful not only for biliary disease, but also for guidewire insertion under direct visualization, thanks to improved visibility.

Endoscopy_UCTN_Code_CCL_1AB_2AZ_3AB

## Notes

Endoscopy E-Videos https://eref.thieme.de/e-videos
*E-Videos* is an open access online section of the journal *Endoscopy* , reporting on interesting cases and new techniques in gastroenterological endoscopy. All papers include a high-quality video and are published with a Creative Commons CC-BY license. Endoscopy E-Videos qualify for HINARI discounts and waivers and eligibility is automatically checked during the submission process. We grant 100% waivers to articles whose corresponding authors are based in Group A countries and 50% waivers to those who are based in Group B countries as classified by Research4Life (see: https://www.research4life.org/access/eligibility/ ). This section has its own submission website at https://mc.manuscriptcentral.com/e-videos .

## References

[1] Lillemoe KD Cameron JL Hardacre JM Is prophylactic gastrojejunostomy indicated for unresectable periampullary cancer? A prospective randomized trial. Ann Surg 1999 230 322 328discussion 328–330 10493479 10.1097/00000658-199909000-00005 PMC1420877

[2] Jeurnink SM Steyerberg EW van Hooft JE Surgical gastrojejunostomy or endoscopic stent placement for the palliation of malignant gastric outlet obstruction (SUSTENT study): a multicenter randomized trial Gastrointest Endosc 2010 71 490 499 10.1016/j.gie.2009.09.042 20003966

[3] Itoi T Ishii K Ikeuchi N Prospective evaluation of endoscopic ultrasonography-guided double-balloon-occluded gastrojejunostomy bypass (EPASS) for malignant gastric outlet obstruction Gut 2016 65 193 195 10.1136/gutjnl-2015-310348 26282674

[4] Chan SM Dhir V Chan YYY Endoscopic ultrasound-guided balloon-occluded gastrojejunostomy bypass, duodenal stent or laparoscopic gastrojejunostomy for unresectable malignant gastric outlet obstruction Dig Endosc 2023 35 512 519 10.1111/den.14472 36374127

